# Morphometric and mechanical characteristics of *Equisetum hyemale* stem enhance its vibration

**DOI:** 10.1007/s00425-017-2648-1

**Published:** 2017-01-07

**Authors:** Urszula Zajączkowska, Stanisław Kucharski, Zdzisław Nowak, Kamila Grabowska

**Affiliations:** 1 Department of Forest Botany, Faculty of Forestry, Warsaw University of Life Sciences, 159 Nowoursynowska St., 02-776 Warsaw, Poland; 2 Department of Mechanics of Materials, Institute of Fundamental Technological Research, Polish Academy of Sciences, 5B Pawińskiego St., 02-106 Warsaw, Poland; 3 Institute of Aeronautics and Applied Mechanics, Warsaw University of Technology, 24 Nowowiejska St., 00-665 Warsaw, Poland

**Keywords:** Mechanical properties, Plant biomechanics, Segmented structure, Stem vibration, Stress distribution, Wind

## Abstract

**The order of the internodes, and their geometry and mechanical characteristics influence the capability of the** ***Equisetum*** **stem to vibrate, potentially stimulating spore liberation at the optimum stress setting along the stem.**

*Equisetum hyemale* L. plants represent a special example of cellular solid construction with mechanical stability achieved by a high second moment of area and relatively high resistance against local buckling. We proposed the hypothesis that the order of *E. hyemale* L. stem internodes, their geometry and mechanical characteristics influence the capability of the stem to vibrate, stimulating spore liberation at the minimum stress setting value along the stem. An analysis of apex vibration was done based on videos presenting the behavior of an *Equisetum* clump filmed in a wind tunnel and also as a result of excitation by bending the stem by 20°. We compared these data with the vibrations of stems of the same size but deprived of the three topmost internodes. Also, we created a finite element model (FEM), upon which we have based the ‘natural’ stem vibration as a copy of the real object, ‘random’ with reshuffled internodes and ‘uniform’, created as one tube with the characters averaged from all internodes. The natural internode arrangement influences the frequency and amplitude of the apex vibration, maintaining an equal stress distribution in the stem, which may influence the capability for efficient spore spreading.

**Electronic supplementary material:**

The online version of this article (doi:10.1007/s00425-017-2648-1) contains supplementary material, which is available to authorized users.

## Introduction

The present-day representatives of the genus *Equisetum* are considered to be survivors from the Devonian period (Husby 2013). Therefore, it comes as no surprise that the 15 species which have survived to this day are subjects of interest for botanists of almost all specializations (Tschudy 1939; Spatz et al. 1998; Marais et al. 2003; Gierlinger et al. 2007; Channing et al. 2011). *Equisetum hyemale* L. is a unique representative of this plant group, primarily due to its special biomechanical features achieved with a simple body structure. It consists of an unbranched column—a stem which attains the height of 150 cm, while the base has a width of 4–6 mm and consists of characteristic ridged internodes and nodes (Niklas 1989b; Speck et al. 1998). The light construction is achieved by the large, central pith cavity and the vallecular and carinal canals. The strengthening tissue consisting of a thick-walled sclerenchyma occurs in the outer part of the perimeter. The central canal qualifies horsetails as a hollow tube structure, in which its geometry and the wall Young’s modulus will significantly determine the mechanical properties of the entire plant, and the transverse nodal septa will increase the resistance of the stem to bending and twisting (Niklas 1997a). Additionally, compared to other hollow tube plants (e.g., grasses) *E. hyemale* is characterized by special anatomical and physiological features that enable it to resist their mechanical collapse because of changes to its cellular water status during the vegetation period. One of these features is the ability to resist sub-freezing temperatures, due extracellular freezing phenomena (Niklas 1989a), whereby shoots remain erect after thawing processes. The second feature is almost independent of hydration state and relates to the ability of shoots to resist local buckling, because of the presence of a double layer of endodermis (Spatz et al. 1998; Speck et al. 1998).

The length of horsetail stem internodes, as well as their width and strength properties varies from the base to the plant apex. Internodes at the base and in the apical region of the stem are shorter and thinner than those in the middle part of the plant. It has also been demonstrated that *E. hyemale* L. internodal diameter and its wall thickness with respect to the internode radius increase from the base of shoots. The high second moment of inertia, from which the stem flexural rigidity results, is the outcome of the distribution of the area and strengthening of tissues in the outer perimeter, and the maximum of this value is located at 1/3 of the plant’s height (Niklas 1989b). Therefore, anatomy, as well as morphometric stem structure parameters, which are variable depending on the stem height, tends toward an increase in mechanical stability. The horsetail is a primitive plant and is not capable of dynamic mechanical adaptation via the formation of specialized tissues typical of specific biomechanical conditions (e.g., reaction wood), of which the higher plants are capable, but its reaction consists in an allometric change of the proportions of individual body parts (Niklas 1989c).

However, morphometric and biomechanical studies on *E. hyemale* do not provide an answer to the question of whether variable internode length and their strength parameters increase stem vibration capability, which, for a plant bearing the strobilus on its apex, may be important for the spore spreading process. Wind, and the oscillation motions induced by it, constitute an integral element creating thigmomorphogenesis in plants (Jaffe 1973; Doaré et al. 2004). This also applies to the type of anatomical adaptation influencing its mechanical characteristics (Niklas and Speck 2001; Gardiner et al. 2016). Vibration of anatomical elements enhancing pollen and spore dispersal with the wind is often a sign of plant adaptation for the optimal use of the physical characteristics of wind for the most efficient dispersal. As an example, it has been demonstrated that inflorescence vibration in *Plantago* is an indispensable factor for pollen release (Urzay et al. 2009; Timerman et al. 2014), similar to sporophyte vibration in certain bryophytes, which aids more efficient spore release (Lee 2010; Johansson et al. 2014); however, there are no studies on the vibration of entire stems in such phylogenetically important plants as *Equisetum.* In such small particles as spores, movement is mostly dependent on drag based on Stokes’s law of resistance (Stevens et al. 1975; Dickinson and Preece 1976; Murray 1986; de Langre 2008). Thus, the dominant factor influencing spore dispersal is wind.

In the horsetail, in which the strobilus is located at the top of the plant, the ejection force resulting from stem flexibility might not be the significant element in spore liberation, but rather that could be the duration vibration of the top portion of the stem, i.e., prolonged exposure to the effect of wind. Our study aimed to demonstrate that the distribution of internode sizes along the stem, as well as their strength properties increase the capability and duration of vibration, thus prolonging the duration of spore dispersal. To test this hypothesis, we performed experiments with static stem excitations of intact stems and stems where the uppermost internodes had been cut. We analyzed their stem vibrations in a wind tunnel, as well spore liberation efficiency. Based on mechanical tests we were able to obtain the stem physical characteristics needed for setting an finite element model (FEM) model that could be transformed to test the morphometric stem parameters essential for the most efficient vibration with the lowest possible mechanical stress.

## Materials and methods

### Plant material

One-year-old *E. hyemale* L. plants with developed strobili were collected from a forest within the Białobrzegi Forest District in central Poland 51°39′46.8″N 20°40′06.3″E at two dates: 26 Sept. 2015 and 5 Oct. 2016. Plants were dug out with rhizomes and placed in pots. The plants were automatically hydrated and stored outdoors at the University Garden, at average daily temperatures of +15 °C (in 2015) and 13 °C (in 2016) according to the weather station at the Warsaw University of Life Sciences.

### Mechanical tests

The mechanical properties of the whole stems of ten plants (height between 476 and 767 mm) were specified in a 3-point bending test of every internode and node. The test was performed using a specially adapted microindentation instrument (Kucharski and Mróz 2007). The samples were placed on supports made of 4-mm-diameter steel cylinders. A similar cylinder was used as an actuator to apply the load at the midpoint of the specimen. The displacement, *f*, of the actuator and the loading force, *P*, were measured using fiber optic non-contact displacement sensors (processing of emitted and reflected light) and a strain gauge, respectively. The load–displacement (*P*–*f*) curves of the actuator were continuously measured. For three-point bending, according to the elastic beam theory, the load displacement relation should have the form of straight line with inclination *k*:

1 $$ \frac{{{\text{d}}P}}{{{\text{d}}f}} = \frac{48EJ}{{L^{3} }} = k, $$

where *L* is the span of the beam, *E, J* are Young’s modulus and moment of inertia, respectively.

First, an analysis of internodes was performed where we had a constant beam section and the direct use of relations (1) was possible. Secondly, analysis was conducted where the stiffness of nodes had to be determined, and beams composed of nodes and adjacent internodes were tested and a more complex approach was required. It was assumed that for rigid beams connected with a compliant node there was a linear relation between the angle of rotation *φ* and bending moment *M* in the node

2 $$ M = b\varphi , $$

3 $$ \varphi = \frac{{f_{\text{r}} }}{L}, $$

where *f*_r_ is deflection in the node, *b* is the stiffness of the node, *L* distance between the support and the node.

**Fig. 1:**
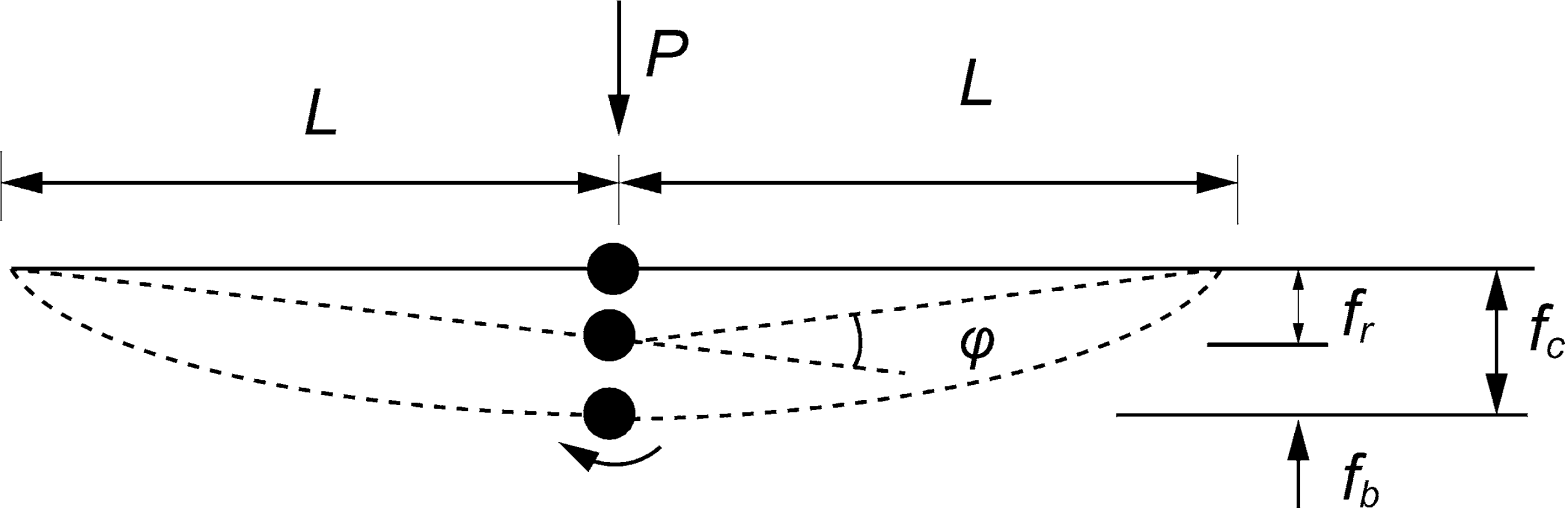
The components of the deflection of a sample with a node in three-point bending. The deformation of the sample with the node is considered a superposition of a compliant beam with a rigid node (deflection *f*_b_) and a rigid beam with a compliant node (deflection *f*_r_, due to rotation in node). This approach enables specification of the stiffness of a node. *L* the span of the beam, *P* loading force, *φ* rotation, *M* bending moment in the node

The deflection measured in a bending test of two internodes connected with a node is the sum of the deflection *f*_r_ of virtual rigid beams due to the presence of angle *φ* in the node and the deflection resulting from bending of the adjacent internodes (actual, compliant beams) *f*_c_ = *f*_r_ + *f*_b_ (Fig. 1).

Coefficient *b* that is a measure of the stiffness in a node can be calculated from the equation:

4 $$ b = \frac{{PL^{2} }}{{2\left( {\frac{P}{{k_{\text{c}} }} - \frac{{PL^{3} }}{12}\left( {\frac{1}{{E_{1} J_{1} }} + \frac{1}{{E_{2} J_{2} }}} \right)} \right)}} = \frac{{L^{2} }}{{2\left( {\frac{1}{{k_{c} }} - \frac{{L^{3} }}{12}\left( {\frac{1}{{E_{1} J_{1} }} + \frac{1}{{E_{2} J_{2} }}} \right)} \right)}}, $$

where *E*_1_ *J*_1_ *, E*_2_ *J*_2_ are the bending stiffness of neighboring internodes, *2L* is the distance between beam supports and *k*_c_ is the tan of the inclination angle of the loading curve and can be specified from the registered *P*–*f* diagram. After the mechanical tests, the length and width of the internodes was measured, as well as the surface area of all the tissues, by cutting an internode in the middle of its length and scanning the cross section with an Epson (Perfection v700 Photo, Tokyo, Japan) scanner at a resolution of 2400 DPI. The obtained images were binarized in ImageJ (Schneider et al. 2012) and the surface area was calculated. Based on the dimensions and weight of an internode, its density was calculated. Using the BoneJ (Doube et al. 2010) plugin, the moment of inertia (*J*) in the examined cross sections was calculated.

### Vibration caused by static excitation

Twenty horsetail stems were deflected from the vertical by approximately 20°, which was chosen as the maximum deflection with no risk of shoot failure. Stem bases were mounted onto a laboratory stand with softly isolated clamp. The second uppermost node was bent to 20° (the angle was drawn on the background wall) and held by a grip mounted to the stand. After releasing the grip, the behavior of the stems was recorded using a Sony RX 100 IV camera (Sony, Tokyo, Japan) at 500 frames per second, adjusting the lens axis perpendicularly to the plant axis. Tests were conducted on intact stems and stems from which the last three internodes had been removed. Stems selected for cutting were initially longer than intact stems, by the length of approximately three internodes. The vibration frequency of the apex and amplitude changes during the vibration absorption were analyzed in Tracker software (https://www.cabrillo.edu/dbrown/tracker/) based on the Open Source Physics (OSP) Java framework. Data were analyzed with a periodogram which shows the main frequencies and their power expressed in units proportional to the square of the amplitudes of sinusoids present in the data (Press et al. 1992). For this analysis, PastProject (Hammer et al. 2001) software was used.

### Spore liberation during stem static excitation

In this experiment the efficiency of spore release was compared between intact stems and stems with the three uppermost internodes cut. For the variant with a cut apical part longer stems were chosen, so that after cutting they had the same height as the intact stem. Due to the high variability of strobilus dimensions and the number of spores inside them, we used small plastic cups with a volume of 20 mm^3^ attached to the apical part of the stem with non-toxic soft modeling clay (Amos, Seoul, Korea) and we filled them full of spores that had been collected (22 Sept.–5 Oct. 2016) from plants growing at the test area (Bialobrzegi Forest Distinct). The cups with spores weighed 1.5 g which was similar to an average strobilus from the tested plants. This was applied for both types of examined stems. The intact stem (with all internodes) had to have its strobilus removed. Horsetail stems were mounted in laboratory stands with elastic and softly isolated clamps and then placed precisely 1 m under a green laser pointer (Wicked Lasers, Kowloon, Hong Kong) positioned parallel to the plant axis in a dark room. The laser pointer generated a multi pattern of light beams (100 mW power output and 532 nm wavelength) that created a pattern of 2 × 2 cm grid points on the base where stems had been placed. Stems were deflected from vertical orientation by 20°, in the same manner as in studies on stem vibration caused by static excitation (mentioned above). After releasing the grip, the behavior of the stems was photographed with 10-s exposure time using a Canon 5d mark IV camera (Canon, Tokyo, Japan) mounted on a tripod.

Three minutes after stem excitation the area about the stem was illuminated by a Blak-Ray B-100Y UV lamp (UVP, LLC, Upland, CA, USA), and the area where spores were visible was measured. The experiment was repeated 11 times for every variant.

### Wind tunnel

Horsetail clumps in pots were subjected to a controlled wind speed effect in a wind tunnel at the Faculty of Power and Aeronautical Engineering at the Technical University in Warsaw. The open-circuit wind tunnel possesses a 1 × 1 m test chamber and generates a maximum flow velocity of 25 m/s. Our study was conducted in a wind velocity range from 0–6 m/s that is similar to the environmental conditions in which the studied horsetail lives (under the canopy in wet deciduous forests) (Geiger et al. 2003). Behavior in the wind was recorded using a Photron Fastcam SA-Z (Photron Limited, Tokyo, Japan) camera at a frame rate of 5000 frames per second. The camera was operated by PFV software (Photron Fastcam Viewer, bundled by Photron together with the camera).

The recorded videos were analyzed with the Tracker software (https://www.cabrillo.edu/dbrown/tracker/) based on OSP which was used to measure:

1. maximum deviation in the *X* axis (perpendicular to main stem axis horizontal line in the image) of the five subsequent stem nodes from the original vertical stem axis (*Y* axis), depending on the wind velocity. Based on video observations, the first 5 uppermost internodes were chosen for analyses given that this zone was dynamically engaged in apex (strobilus) vibration. The analysis was performed on an image of a feature of interest (the boundary between the dark whorl of leaves and the light stem), then by searching each frame for a match to that template. The results are a set of *X*, *Y* coordinates that could be transformed into positional changes along *X* or *Y* axes. Ten stems were analyzed.
2. vibration frequency and amplitude of intact stems in comparison to three cut apex internodes (average 8 cm) with equal height. The amplitude was measured based on the maximum deflection of the measured apex along the *X* axis. During a manual mark, the apex point on the video timeline frame, when the vibration period had ended, and then based on the frame rate per second of the video, the real frequency of the apex was recalculated. Ten intact stems and ten cut stems were analyzed (Supplementary material Video S1). The results were analyzed using a periodogram showing main frequencies.

### Numerical simulation

#### Finite element modeling (FEM)

The numerical simulation of stem oscillation by using FEM modeling allowed us to obtain information both about the kinematic state of its structure at given test stages, and about the continuous deformation process and the corresponding stress fields. In the numerical simulation, three models of stem structures were assumed:

- one stem called the ‘natural’ stem composed of ten segments (internodes) with connectors (nodes), with the same dimensions and properties (Young modulus and bending stiffness) as presented in Table 1,

**Table 1:** Results of measurements of ten internodes and nine nodes of a stem which was the basis for the FEM model in the ‘natural’ version

No. internode	No. node	*L* (mm)	*D*_max_ (mm)	*D*_wall_ (mm)	*E* (MPa)	*J* (mm^4^)	*b* (Nmm)
1		51.44	3.51	0.30	409.77	4.23	
	1						56.43
2		66.34	3.62	0.30	274.20	10.21	
	2						110.66
3		89.99	3.81	0.15	463.40	6.33	
	3						313.98
4		91.96	4.63	0.40	314.40	12.72	
	4						977.47
5		95.69	5.19	0.65	199.10	20.09	
	5						19,226.30
6		86.78	5.18	0.75	166.20	27.26	
	6						11,973.01
7		82.11	5.31	0.75	175.20	31.20	
	7						37,846.15
8		74.13	5.62	0.90	220.00	32.12	
	8						11,210.96
9		65.37	5.18	1.05	304.90	27.11	
	9						2523.08
10		62.78	5.08	1.05	354.10	27.11	

*L* internode length, *D*_*max*_ internode diameter measured in the middle of the length, *D*_*wall*_ wall thickness, *E* elastic modulus, *J* inertia modulus, *b* node stiffness
- two virtual stems—the ‘random’ stem composed of the same segments as for ‘natural’ stems; however, the order of the segments was arbitrarily changed using a random numbers generation procedure. The following order of the internodes was generated: 1, 2, 7, 6, 5, 4, 8, 10, 9 (the internode No. 1 was located at the top and internode No. 9 at base of the virtual stem). The ‘uniform’ stem composed of one segment with the length of a ‘natural’ stem and other dimensions and material properties calculated as an average of ‘natural’ segments.

For the above three kinds of stem structure numerical simulations were performed where Hooke’s law had been assumed. The damping effect was included in the model that is viscoelastic material properties were assumed. The effect of damping was analyzed for two other kinds of stem that were investigated experimentally, as was described in the previous section: the ‘cut’ stem and the ‘intact’ stem. For the latter, the same geometry was assumed as for the ‘natural’ stem.

In viscoelastic materials, the constitutive relations involve quite general, the stress rates and the strain rates. The “VISCOELASTIC, TIME = PRONY” option in Abaqus specifies dissipative behavior for use with elasticity and describes isotropic rate-dependent material behavior for materials in which dissipative losses are primarily caused by “viscous” (internal damping) effects. Abaqus assumes that the viscoelastic material is defined by a Prony series expansion of the dimensionless relaxation.

A numerical simulation was applied to evaluate local stress distribution along the stems and to find potential differences in vibration between these stems, following the hypothesis that frequency is important for spore dispersal, because it influences the velocity and acceleration of stem motion.

### Finite elements mesh

Volumetric meshing uses the natural discretization of an 8-node cubic element. The element type is C3D8 from the Abaqus commercial code (Simulia 2013). This is an eight-node brick element with linear interpolation. Such first-order elements capture stress concentrations and are effective in bending-dominated problems. Each stem is composed of ten cylindrical segments and nine cylindrical connectors. Each segment has a different diameter, length and thickness. The connectors have the form of short (2 mm) cylinders with variable thickness, and the thickness at each end of a connector equals the thickness of the adjacent segment. The stiffness of connectors corresponds to the stiffness of nodes specified in the experiment. At the interface between internode and node, the “tie” option included in the Abaqus software was used. This makes the translational and rotational motion as well as all other active degrees of freedom equal for a pair of surfaces in contact. In numerical simulations, the total number of elements and the total number of nodes in the ‘natural’ stem model is 4224 and 9313, in random stem models 3776 and 8768 and in uniform stem models 3200 and 6464, respectively.

### Boundary and initial conditions

In all considered models the bottom end of the sample is clamped, and the upper end is constrained so that only a motion in one (*X*–*Y*) plane is possible. We assumed that this simplification of the actual motion of stems observed in the wind tunnel still enabled a fair comparison of the motion of different stems and did not influence the conclusions presented in the paper. We also assumed free lateral faces. The initial angular velocity on the upper five segments was 0.34 rad/s, the assumed velocity enabled in the first half-cycle, at the beginning of the process, the same displacement amplitude at the top of the stem as in the mechanical test (deflection from the vertical by approximately 20°).

### Anatomical observations

The surfaces of the horsetail stems, as well as their cross sections were visualized using scanning electron microscopy (Fei Quanta 200; Thermo Fisher Scientific, Waltham, MA, USA) at 25 kV. Microscopic observations of stem anatomy were performed on transverse and tangential sectional samples of stems in an Olympus BX-61 (Tokyo, Japan) optical microscope using UV light. Specimens were fixed in formaldehyde:acetic acid:50% ethanol (FAA; 1:1:18, by vol.), dehydrated with ethanol, embedded in epoxy resin (Epon 812, Serva, Sigma-Aldrich, St. Louis, MO, USA), then cut in sections of 5–8 μm using a Leica UC7 ultramicrotome (Leica Microsystems, Wetzlar, Germany) and stained with an aqueous 1% safranin solution.

## Results

### Mechanical tests

**Fig. 2:**
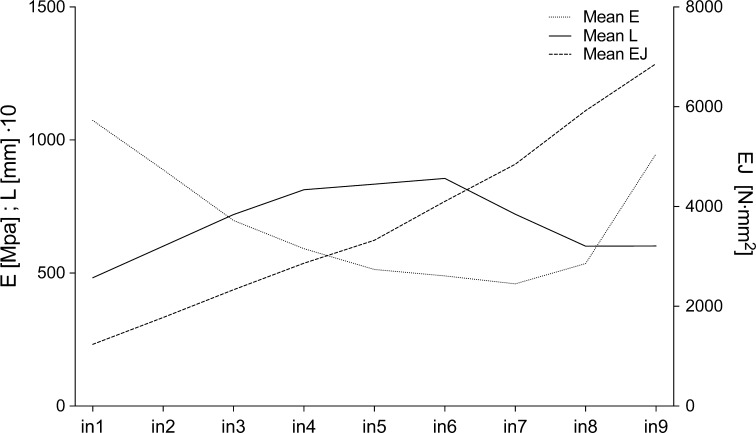
Modules of flexibility (*E*) and bending stiffness (*EJ*) of internodes (*in*) and their length (*L*) for ten tested plants (*points*). *Lines* represent means from ten measurements. A variable internode length is visible, with bending stiffness decreasing with stem height, and flexibility modules are at their highest levels at the basis (*in9*) and at the top (*in1*) of the stem

**Fig. 3:**
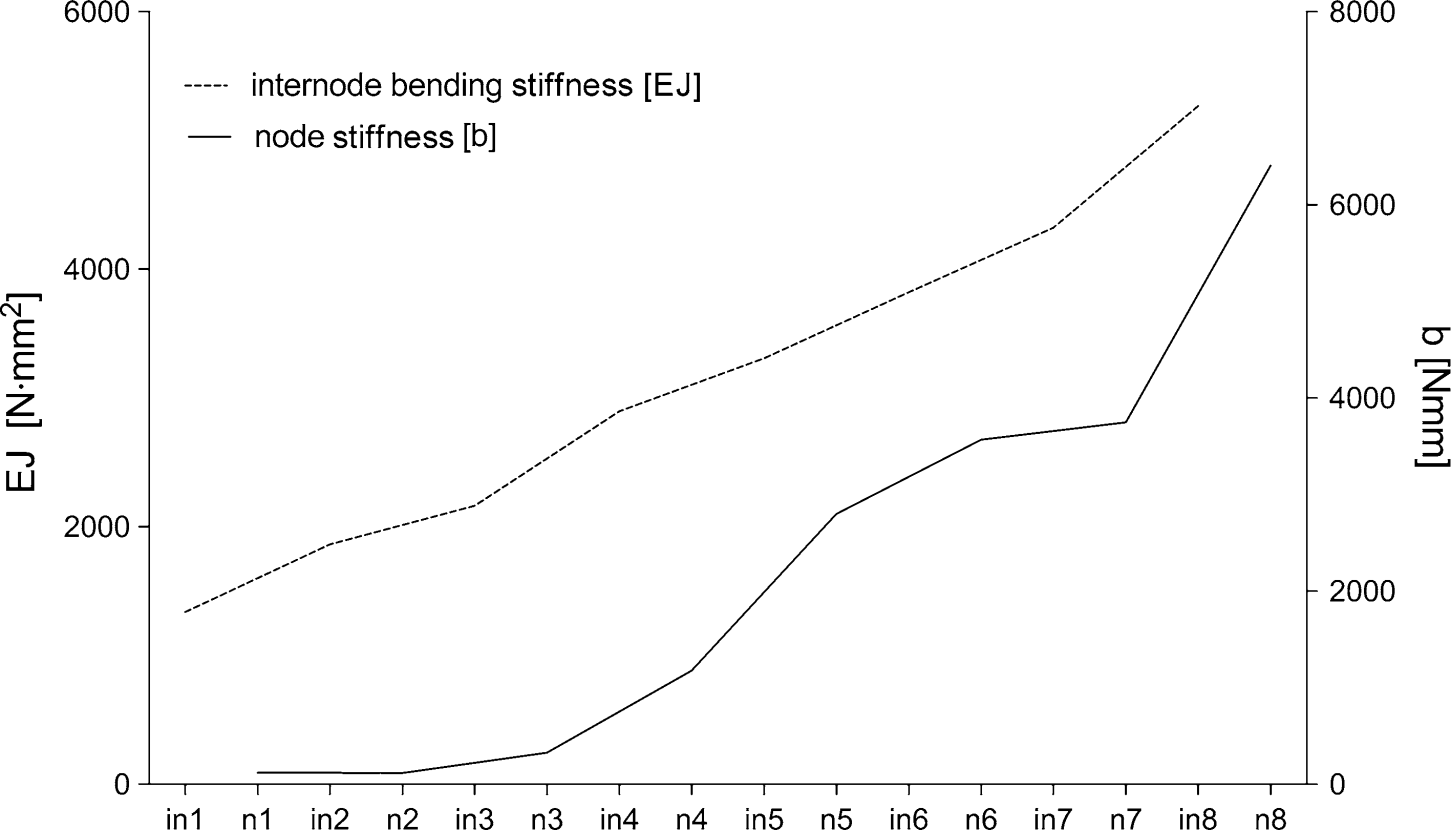
Internode bending stiffness (*EJ*) and node stiffness (*b*) distribution in subsequent nodes and internodes. Means from ten stems. Increased stiffness of stem elements from its lower portion is visible

The analyzed plants exhibited stem internode lengths typical for horsetails, i.e., the shortest internodes were found at the top of the stem and at its base, and the longest internodes were located in its middle portion (Fig. 2). Bending stiffness (*EJ*) increased from the apex to the base, flexibility modulus (*E*) was the lowest in the middle of stem. The node stiffness (*b*) was greater in the lower half of the stem; however, this character increased considerably, reaching values up to fivefold higher than those of nodes at the top (Fig. 3).

### Vibration caused by static excitation

**Fig. 4:**
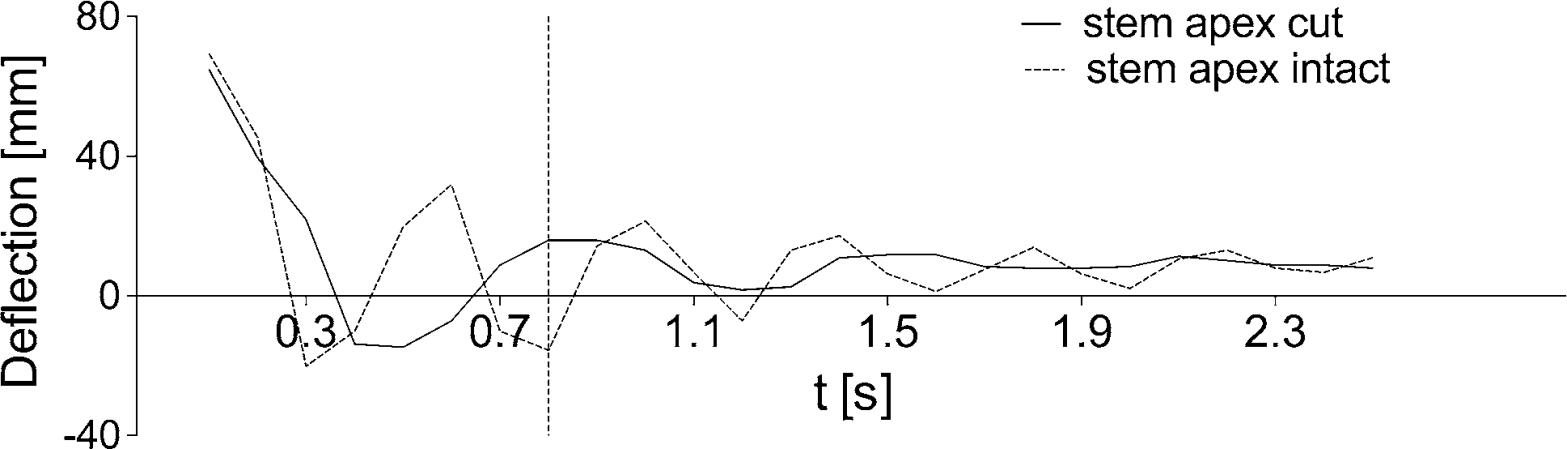
Comparison of intact stem vibration and vibration of stems with the three top internodes cut off, at equal height (62 cm), after static excitation via deflecting the stem by 20° from the vertical. Vibrations of the shortened stem are dampened almost instantly. Intact stem vibration has a higher frequency and is dampened at a later time. The chart covers a period of 2.5 s

After deflecting the intact stem by 20°, it vibrated with a frequency of approximately 1 Hz, and after approximately 2.5 s a clear reduction of vibration occurred. In every case, a stem with the same length, but with the three top internodes removed showed lower amplitude of vibration and stopped approximately after 1.5 s (Fig. 4; Supplementary material Fig. S1a, b). Frequencies measured from excitation to end of movement showed that intact stems showed a two times higher frequency (2.4 Hz) than stems with the apex cut (1.2 Hz) (Supplementary material Fig. S1c).

### Spore liberation during stem static excitation

**Fig. 5:**
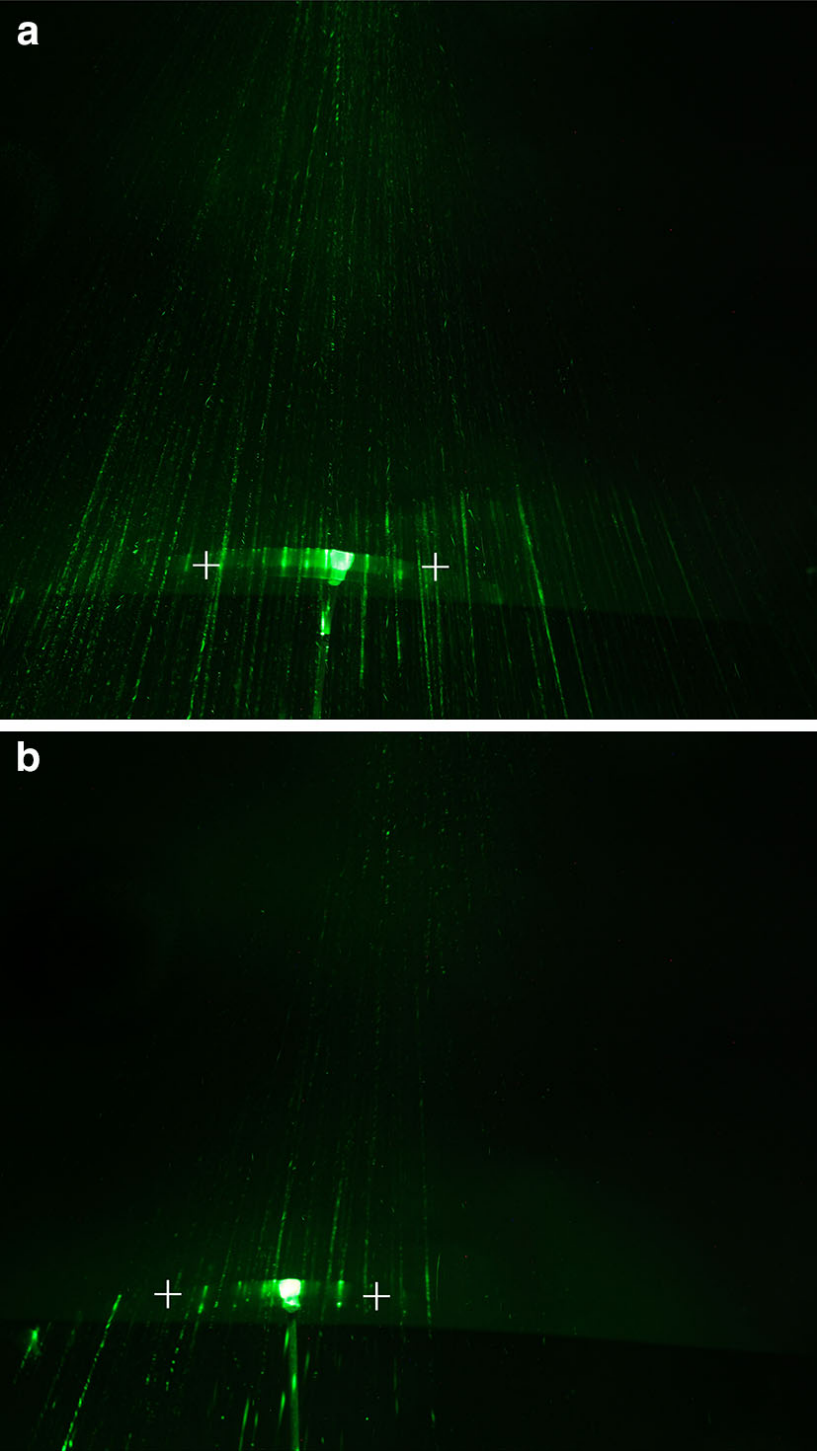
Spore liberation during static excitation of intact stems (**a**) and stems with three uppermost internodes cut off (**b**). Images taken with 10-s exposure time. Maximal deflection points of the apex are marked by *white crosses*. Released spores light at the moment of laser beam crossing. Intact stem vibrates longer and the time of dispersion of spores makes it more efficient. It is clear that more lightening points were taken during exposition time

After excitation the stems vibrated and spore liberation was recorded for 10 s. In clear and dry air the laser beam was invisible, when a spore crossed the laser line a light sparkle was observed (Supplementary material Video S2). Intact stems released significantly more spores on a larger area compared to stems without the three uppermost internodes (Fig. 5).

The measured area of spores distributed around the stem showed that an intact stem released spores over an area two times greater (mean 1179 cm^2^, standard deviation 316 cm^2^) than the cut stems (mean 507 cm^2^, standard deviation 260 cm^2^).

### Wind tunnel

**Fig. 6:**
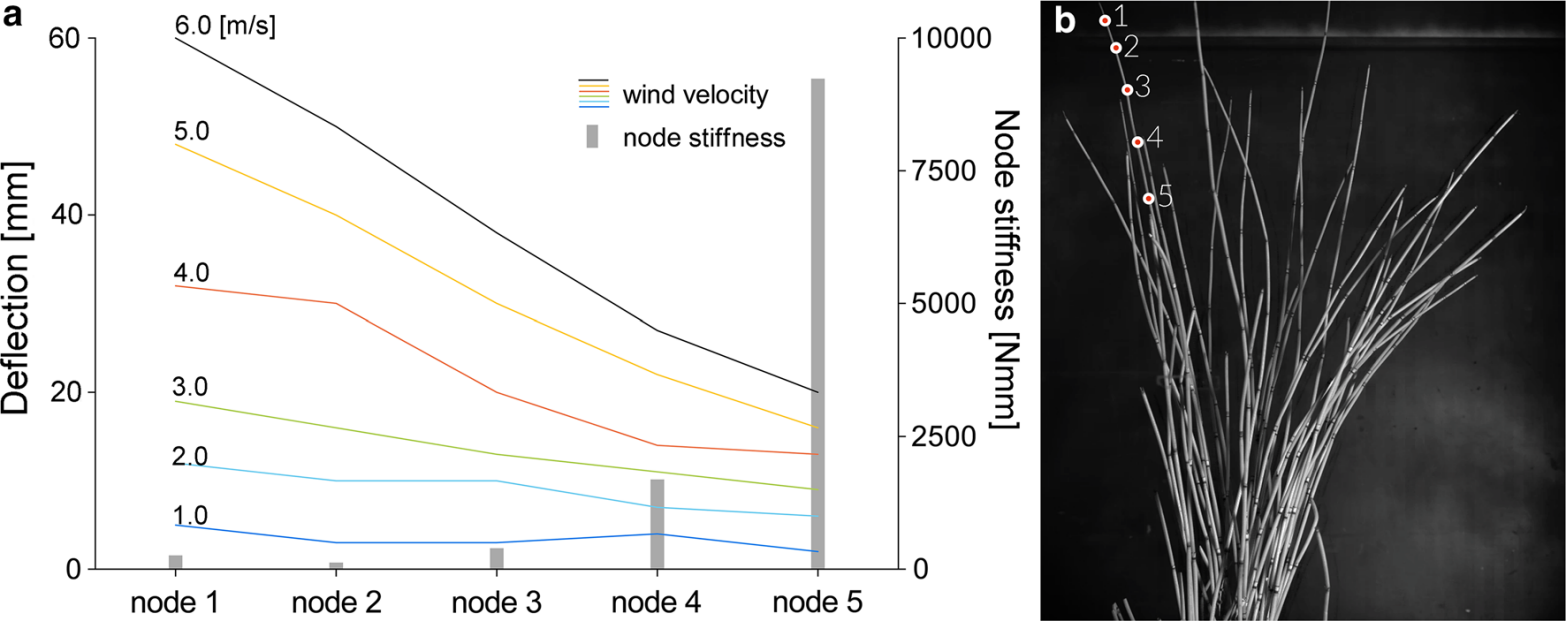
Deflections of five nodes filmed in a wind tunnel depending on the wind speed and node stiffness. **a** The stem vibration susceptibility decreases with higher node stiffness, which can be seen based on the example of the three uppermost nodes, which exhibited the highest deflection and very low stiffness. **b** Frame from the video presenting the studied *Equisetum* clump with analyzed internodes marked (*red points*)

**Fig. 7:**
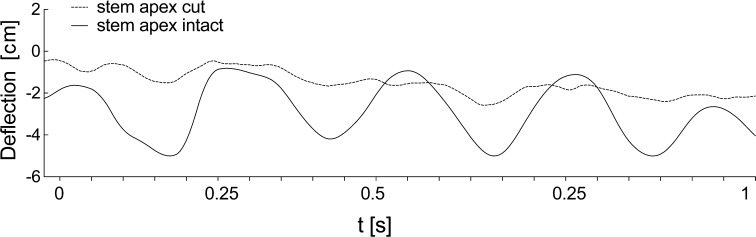
Comparison of vibrations of intact stems and with first three uppermost internodes cut to an equal height (67 cm) and tested in a wind tunnel (fragment from 1-s measurement) at a velocity of 6 m/s. Vibration of the shortened stem is very low and irregular. The intact stem vibration has a higher frequency and regularity

Horsetail stems subjected to the effect of wind increase their stem deflection depending on wind velocity, which shows that up to a velocity of 3 m/s, the stems vibrate with relatively low deflections (1.9 cm for node 1) (Fig. 6). A twofold increase in the velocity, to 6 m/s, caused a threefold increase in deflections (6 cm for node 1). Additionally, for this velocity it was observed that the nodes may hit against each other without any influence on stem deflection but rather only on the rearrangement of the initial vibration state. The capability for deflections for each part of the stem is strongly linked to node stiffness. Our analyses demonstrated that the zone significant for the vibration process is the height marked by the three top nodes, which was confirmed by the analysis of the vibration of stems with three apex nodes cut off. In comparison to the intact nodes with the same length, where deflections reached 5 cm during vibration, the shortened stems moved with lower deflections (by a maximum of 1.5 cm) (Fig. 7; Supplementary material Fig. S2a, b). The vibration of stems without the uppermost internodes showed lower amplitudes and a lower range of frequencies. Peaks of dominant frequencies for intact stems were about 6 Hz and for cut stem three dominant peaks (approximately 2, 3 and 5 Hz) occurred. These observations were confirmed using the periodogram plot (Supplementary material Fig. S2c).

### Numerical simulation

The vibrations of stems were analyzed numerically over the time period of about 3 s, which corresponds to several oscillations. The deflection of each point of stems was calculated for each time point. We assumed that the minimization of stress is one of the criteria for the optimal “design” of stems. Therefore, as the stress state is not uniaxial, the von Mises equivalent stress

$$ \bar{\sigma } = \frac{1}{\sqrt 2 }\sqrt {(\sigma_{1} - \sigma_{2} )^{2} + (\sigma_{2} - \sigma_{3} )^{2 } + (\sigma_{3} - \sigma_{1} )^{2} } , $$

where *σ*_1_, *σ*_2_, *σ*_3_ are principal stresses, was used to present results. For each kind of stem, two specific time points were selected from the whole time history, when a local maximum of equivalent stress occurs.

### Simulation of local equivalent stress maxima during stem deflection

**Fig. 8:**
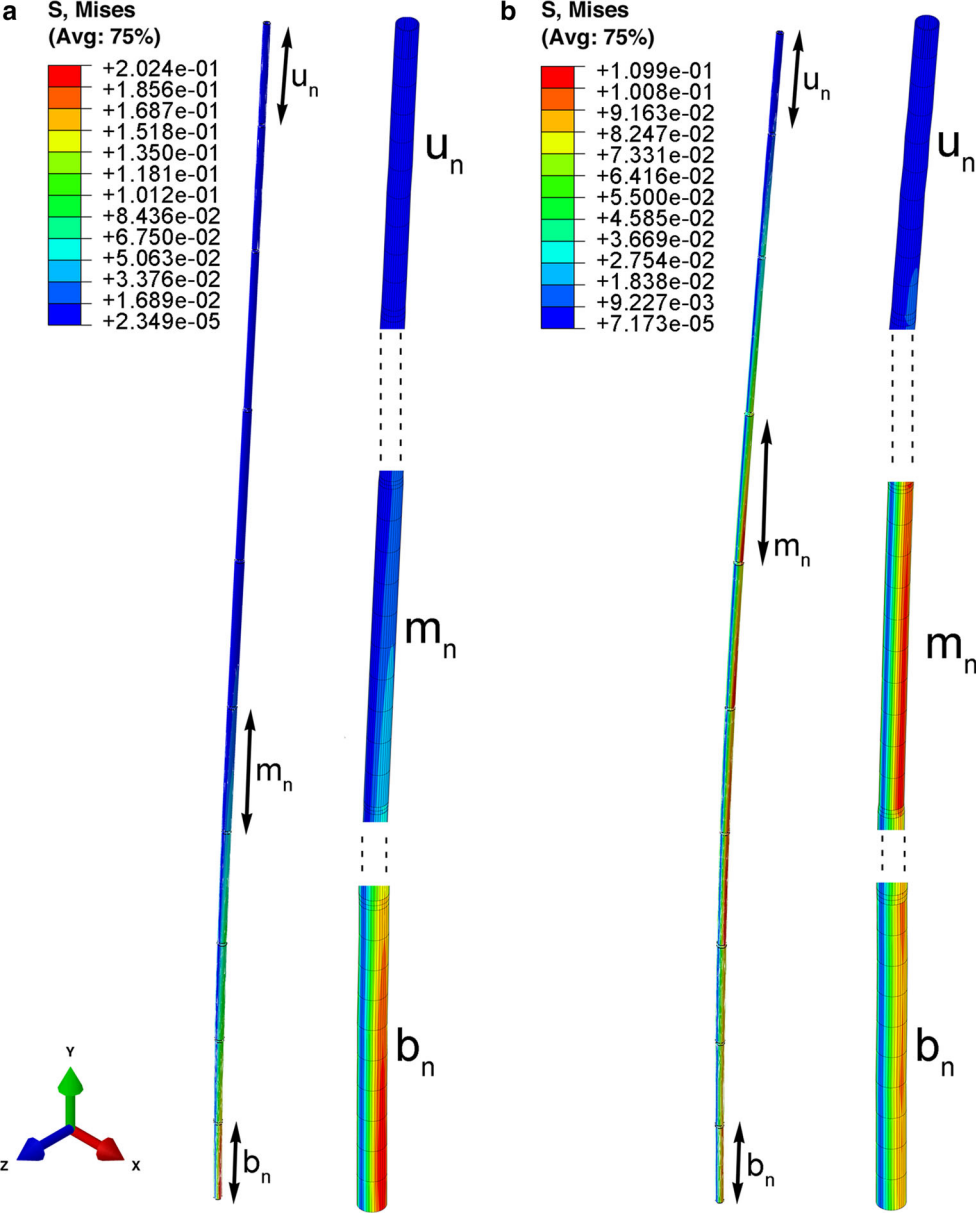
The deformation modes and Mises equivalent stress distribution in a ‘natural’ stem at time points 0.78 s (**a**) and 1.5 s (**b**). The stress maximum is in the bottom of stem and it propagates into the middle part. The total stem length is *L* = 766 mm; the internode length (*l*) and diameter (*D*) are as follows: upper part (*u*_n_) (first internode, *l* = 51.4 mm, *D* = 3.5 mm), middle part (*m*_n_) sixth internode, *l* = 86.78 mm, *D* = 5.4 mm for **a**; and for **b**, fourth internode, *l* = 91.96 mm, *D* = 4.6 mm, bottom part (*b*_n_) (tenth internode, *l* = 62.78 mm, *D* = 5.1)

**Fig. 9:**
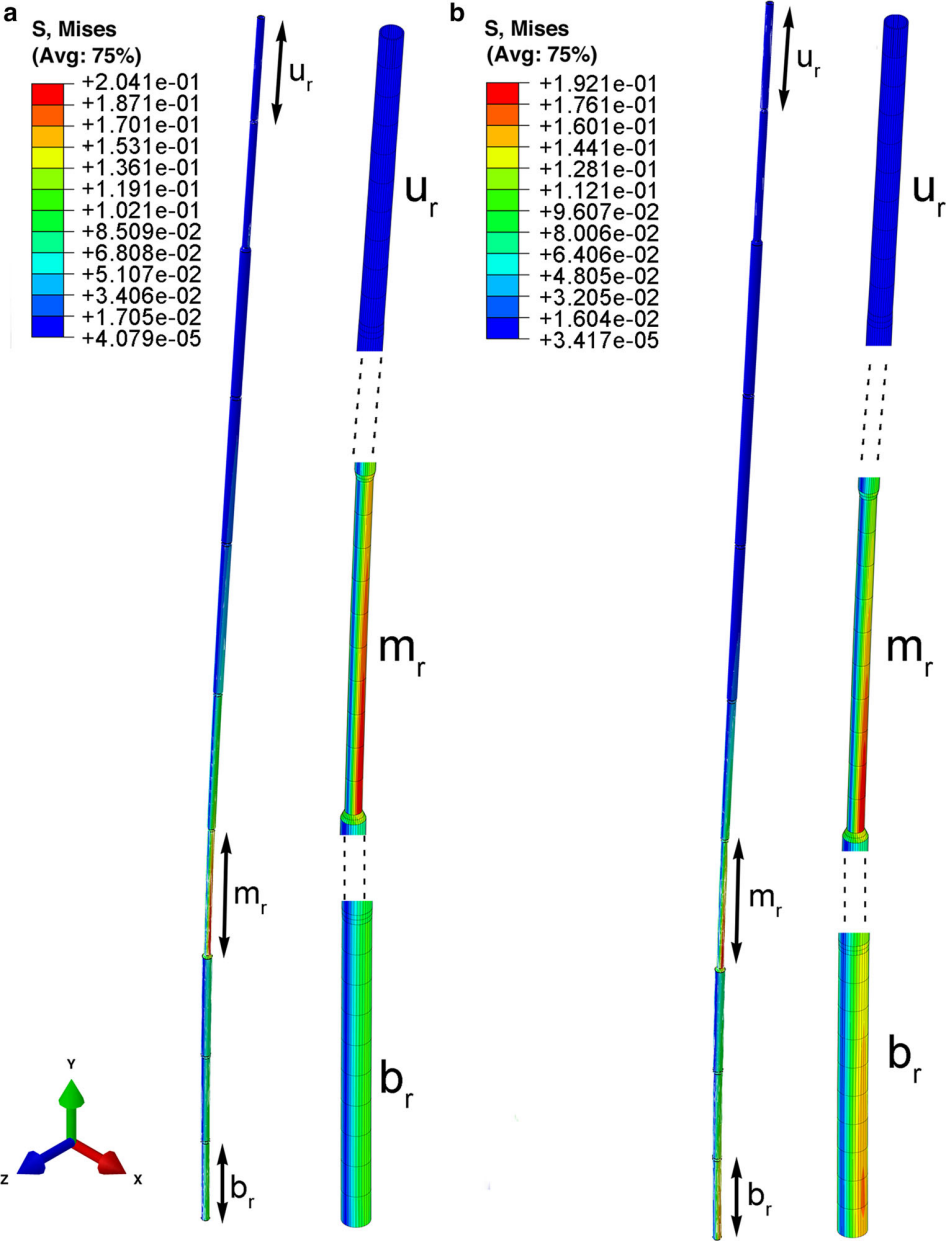
The deformation modes and Mises equivalent stress distribution in ‘random’ stems at time point 0.18 s (**a**) and 0.21 s (**b**). The stress maximum is in the seventh (thinnest) internode: stem length *L* = 766 mm; the internode length (*l*) and diameter (*D*) are as follows: upper part (*u*_r_) (first internode, *l* = 51.44 mm, *D* = 3.5 mm), middle part (*m*_r_) (seventh internode corresponding to the third in a ‘natural’ stem *l* = 89.99 mm, *D* = 3.8 mm), bottom part (*b*_r_) (tenth internode, *l* = 62.8 mm*, D* = 5.1 mm)

**Fig. 10:**
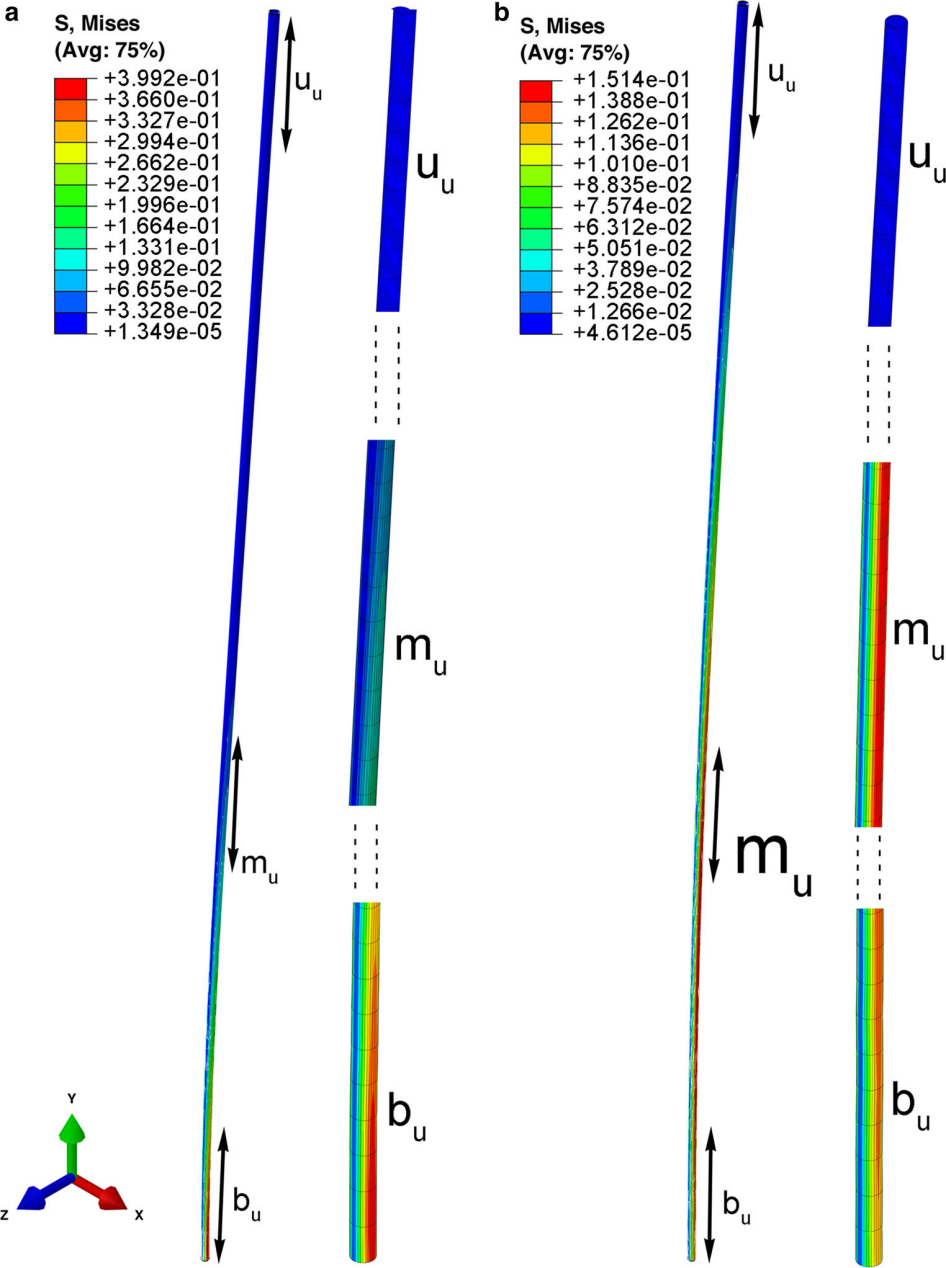
The deformation modes and Mises equivalent stress distribution in ‘uniform’ stems at time points 0.84 s (**a**), and 1.5 s (**b**). The stress maximum is in the bottom part, and then in the middle part. Length of whole stem *L* = 766 mm, *D*_mean_ = 4.73 mm

The local stress maxima were observed in the bottom internode or in one of the middle internodes (near the midpoint of the stem length). In the highest internode, the stress level is always low. The stress states in the bottom, middle and upper regions of the stem corresponding to the selected time points are presented in Figs. 8, 9 and 10. The change of the location of stress maxima during the process results from the superposition of different vibration modes. For ‘natural’ stems, the maximum value of stress (0.202 MPa) at the bottom zone of stem was observed at the 0.78 s time point. Next, at 1.5 s, the second maximum (0.11 MPa) was seen approximately in the middle of the stem height, while at the same time the stress value at the bottom equaled 0.095 MPa (Fig. 8; Supplementary Material Video S3). For ‘random’ stems, the maximal value of stress equaled 0.208 MPa and this was seen at 0.18 s in the middle of the stem, where the thinnest internode is located. The maximum of stress in the bottom of stem can be observed at the time point 0.21 s and this equaled 0.169 MPa (Fig. 9; Supplementary Material Video S4). For ‘uniform’ stems, the maximum stress values (0.4 MPa) were observed at the bottom, at the time 0.84 s. The local maximum (0.15 MPa) in the middle of the stem was observed at the time point 1.5 s (Fig. 10; Supplementary Material Video S5). In the upper region of all stems, the stress level was low during the whole period.

### Simulation of stem vibration

**Fig. 11:**
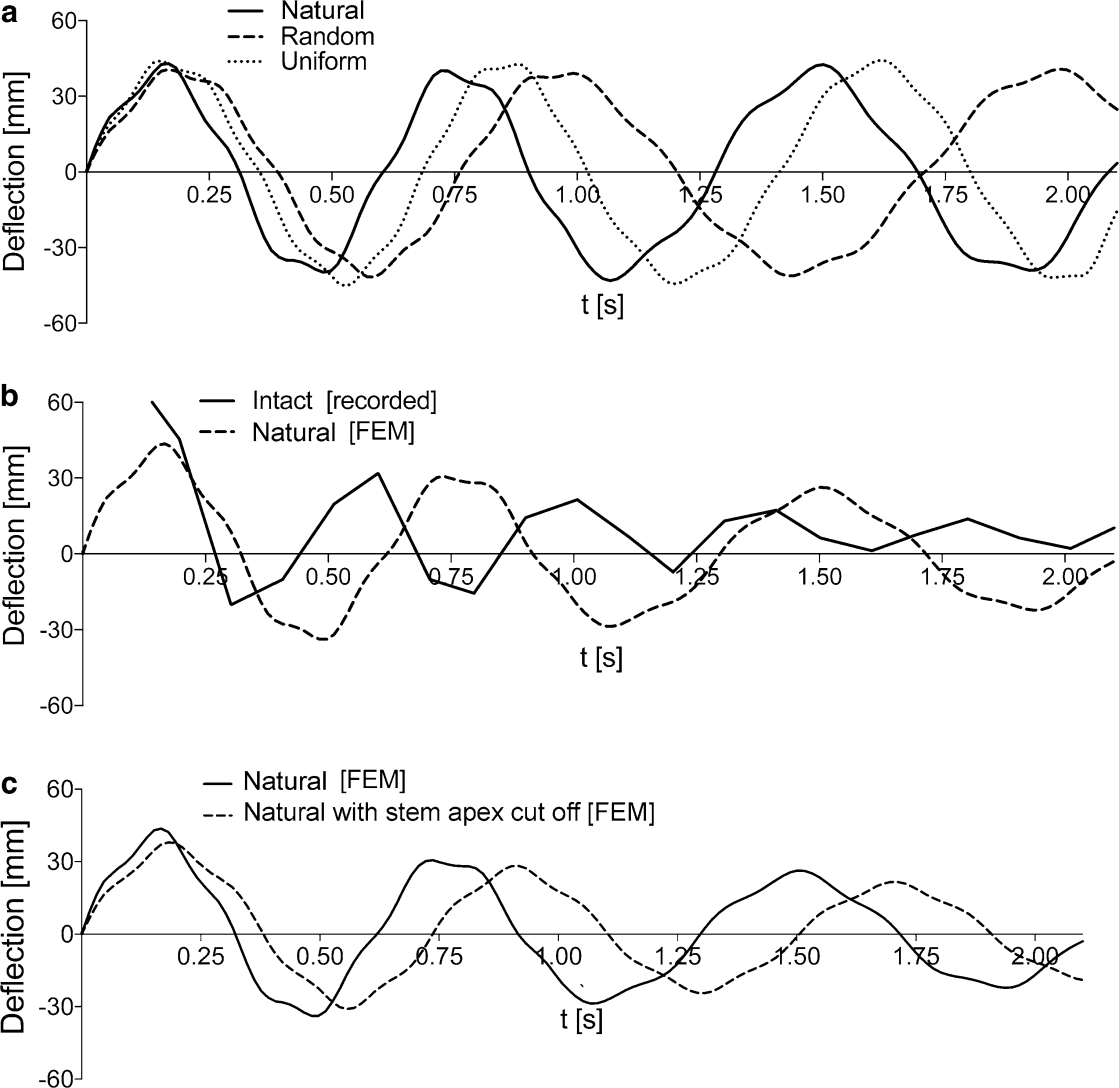
The characteristics of motion of the stem apex as a function of time. **a** Comparison for three considered stem structures—‘natural’, ‘random’, ‘uniform’—generated by a finite element model (FEM) using an elastic material model of a particular stem for 2-s vibrations; ‘natural’ stem vibrations have higher frequencies. **b** Comparison of intact stem (equivalent to ‘natural’ stem) recorded after dynamical excitation, and an FEM model that takes into account the damping effect and that is based on ‘natural’ stem mechanical and morphometric parameters. Close agreement between experimental and numerical results is observed. **c** Comparison of FEM models of ‘natural’ with apex and ‘natural’ with apex cut stems after static excitation, where damping parameters are the same

In numerical simulations both elastic and viscoelastic material models were considered. The model of vibration of the three stem types (‘natural’, ‘random’, ‘uniform’) only includes the frequency sizes without the absorption phenomenon. However, the characteristics of apex deflection of the stem apex as a function of time for the three considered models showed that the highest frequency of apex motion occurred for ‘natural’ stems (Fig. 11a). Vibration amplitudes for the simulated stem did not differ from those recorded for real plants (Fig. 11b) and the earlier presented mechanical deviation from the vertical position (Fig. 4). From a comparison of elastic numerical solutions and experimental results one can notice that in real stems absorption occurs almost from the beginning of vibration.

The absorption phenomenon was considered in the numerical analysis of the ‘intact’ and ‘cut’ stems (Fig. 11b, c). In both cases, the same viscoelastic material parameters were assumed. For ‘intact’ stems, a close agreement between theoretical and experimental results can be observed (Fig. 11b).

## Discussion

**Fig. 12:**
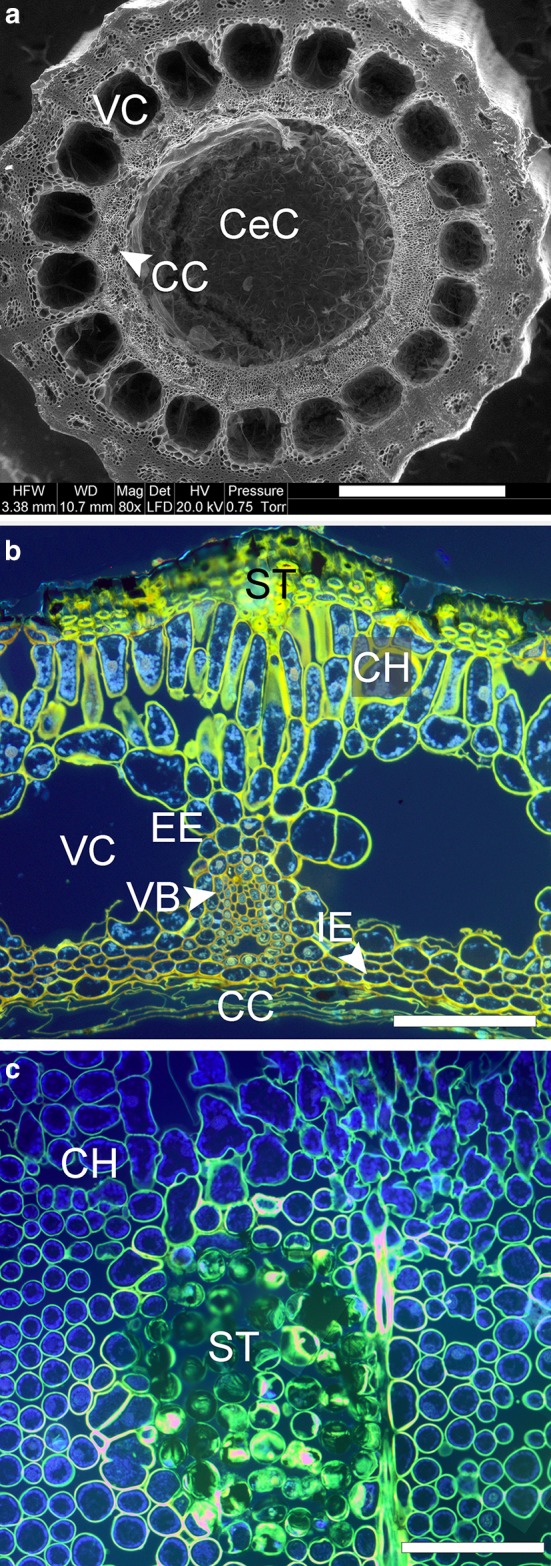
Anatomical structure of *Equisetum hyemale* stem. **a** Cross section of the lower internode of horsetail stem with central pith cavity (CeC), vallecular canals (VC) and carinal canals (CC). SEM photograph. **b** Cross section of internode wall with visible vallecular (VC) and carinal canals (CC), chlorenchyma cells (CH) and vascular bundle (VB), internal endodermis (IE), external endodermis (EE) and strengthening sclerenchyma on the protruding ridges (ST). UV–light photograph. **c** Tangential section of the horsetail stem with visible ordered layer of photosynthetic chlorenchyma cells (CH) and central group of strengthening cells (ST). *Scale bars* 1000 μm (**a**), 100 μm (**b**, **c**)

Our study indicates that only apexes of stems with the natural order of internodes may vibrate for the longest period and have the highest vibration amplitude and frequency, which influences the efficiency of the spore liberation process. The horsetail internodes are shorter at the top, and the nodes have less stiffness, which has already been observed (Niklas 1989b; Spatz et al. 1998; Hogan and Niklas 2003). However, testing whether these characteristics may be of functional importance has not yet been the subject of experiments, apart from the fact that the stems are anatomically and physiologically optimized to increase biomechanical stability. The mechanical and morphometric properties of stems, as well as their anatomy, enable them to achieve the unique capability to perform elastic deflections in the form of apex vibration, probably with lower risk of plastic deformations of the stem. We can speculate that if water transport within metaxylem and carinal canals has low efficiency in *Equisetum* stems which is due to the anatomical features (Carlquist and Schneider 2011), then every deformation within a stem can be a major risk factor for water transport. The existence of strands of sclerenchyma binding the inner and outer parts of the stem constitutes a particularly important adaptation (Fig. 12a–c). Stems with thin walls are susceptible to local buckling during vibrations. Numerical engineering analyses have demonstrated one of the methods of construction reinforcement to protect stems against buckling (Hibbit 1979), which for horsetail is most probably the sclerenchyma and double layer of endodermis (Spatz et al. 1998; Speck et al. 1998).

*Equisetum hyemale* lives in clumps, which causes the stems to hit against each other in the wind, which we have observed in wind tunnel experiments which among others showed that during wind higher deflection is noted in the uppermost internodes. Such a phenomenon may be significant for spore spread, because of changes in the kinetic energy of stems during their collision. Hypothetically, it can be assumed that spores in the vibrating strobilus have the same speed as the strobilus; at the moment of collision with the second stem, the spores are liberated from the strobilus and they may have a slightly lower speed than the speed just before collision.

In structures with hollow tube stems, their nodes operate as elastic springs, which store elastic energy during stem vibration and internode deformation (Niklas 1997b). This energy is immediately released, directly influencing the vibration frequency. This may be an important feature for the horsetail, where there is a clear node stiffness gradient. Indeed, stored elastic energy increases with increasing stiffness; it is higher in the nodes of the middle and lower parts of the stem, and it is released and capable of increasing vibration of the top, light and flexible portions.

The results of our experiments demonstrate that if the stem is deprived of the three top nodes, thus exposing to deflection only internodes connected with nodes of high stiffness, the stem exhibits considerably lower vibration, which is rapidly absorbed (Figs. 4, 7).

Moreover, it appears that a threshold force exists, which is necessary for initiation of the elastic spring phenomenon in nodes. This phenomenon assumes that nodes are able to store and release energy on the stem. The study in the wind tunnel demonstrated that clear deviations of the stem apex during vibrations appear at the velocity of 4 m/s (Fig. 6).

In our study, we created an finite element model (FEM) simulation for the behavior of a ‘natural’ stem, reflecting both morphometric as well as mechanical properties of the natural plant, and a stem with randomly shuffled internodes, as well as a uniform model, i.e., a straight hollow tube without nodes, consisting of one segment being the mean of the dimensions from all internodes. The vibration frequencies were highest in the ‘natural’ stem and, simultaneously, local stresses in the entire stem were the lowest. It should be noted that the lowest value for the local stress maximum is observed for a ‘natural’ stem. Thus, the ‘natural’ stem exhibits an optimal configuration from the point of view of reduction of stress levels after dynamic excitation. This may be important for enhancement of the fatigue life of stem structures when a high cycle loading in vibration process is considered. It should be noted that in the ‘uniform’ stem, with dimensions calculated as a mean value of all ‘natural’ internodes, twofold greater stress values are observed than in the ‘natural’ stem after the same excitation.

In the FEM simulation, the propagation of local stress during vibration can be observed. For the ‘random’ model, accumulation of stress was noted in the inner, thin internode (Fig. 9). Such a strong accumulation of local stress remaining in one zone of the stem is not observed in ‘natural’ stems. These are moved with the structures to the upper parts, and become dispersed to the level of the fourth internode, i.e., where the internode bending stiffness rapidly decreases (Fig. 3). During the entire vibration period, the three uppermost internodes of ‘natural’ stems are vibrating not only with the highest amplitudes, but also entirely free of clear stress gradients. Even though the FEM model was consistent with the general rule that an intact stem vibrates with higher frequency and its damping process occurs for a longer period, we could not obtain results that reflect experimental data (frequency rate and damping period). This is another proof that FEM models should be based on structural complexity and on precise anatomical and micro-biomechanical records.

### *Author contribution statement*

UZ conceived and designed the research, conducted microscopic observations, designed and performed spore liberation experiments, analyzed all data, and wrote the manuscript. SK and UZ conducted measurements and analyzed indentation test data. NZ prepared numerical simulations. DG and UZ performed wind tunnel experiments and analyzed stem vibration data. All authors read and approved the manuscript.

## Electronic supplementary material

Below is the link to the electronic supplementary material.

**Fig. S1** Comparison of the vibration of intact stems. Nine stems with the three top internodes cut off (**a**), and after static excitation via deflecting the stem by 20° from the vertical (**b**). Distribution of dominant frequencies presented in a periodogram for intact stems and apex cut stems (**c**) (TIFF 833 kb)

**Fig. S2** Comparison of the vibration of intact stems. Nine stems with the three top internodes cut off (**a**), recorded in wind tunnel (**b**). Distribution of dominant frequencies presented in a periodogram for intact stems and apex cut stems (**c**) (TIFF 1128 kb)

**Video S1** Deflection of apex during wind tunnel experiment (wind velocity 4 m/s) analyzed using Tracker software on 226 video frames. Green mark on apex shows stem intact, and red with cut uppermost three internodes. In the upper-right window of the Tracker software there is a deflection along the X axis during measured time (0.9 s). In the lower right window coordinates of each point are visible (MOV 9774 kb)

**Video S2** Video presenting spore release in experiments with stem static excitation. Spores crossing laser beam sparkle (MOV 10257 kb)

**Video S3** The dynamics of reduced stress (Mises) during oscillations of the modeled ‘natural’ stems (MOV 2002 kb)

**Video S4** The dynamics of reduced stress (Mises) during oscillations of the modeled ‘random’ stems (MOV 1837 kb)

**Video S5** The dynamics of reduced stress (Mises) during oscillations of the modeled ‘uniform’ stems (MOV 1864 kb)
