# Supplementary figures and images for: Morphometric and mechanical characteristics of Equisetum hyemale stem enhance its vibration
Source: Planta. 2017 Jan 7;245(4):835–48. doi: 10.1007/s00425-017-2648-1 (PMC5357508; doi:10.1007/s00425-017-2648-1)

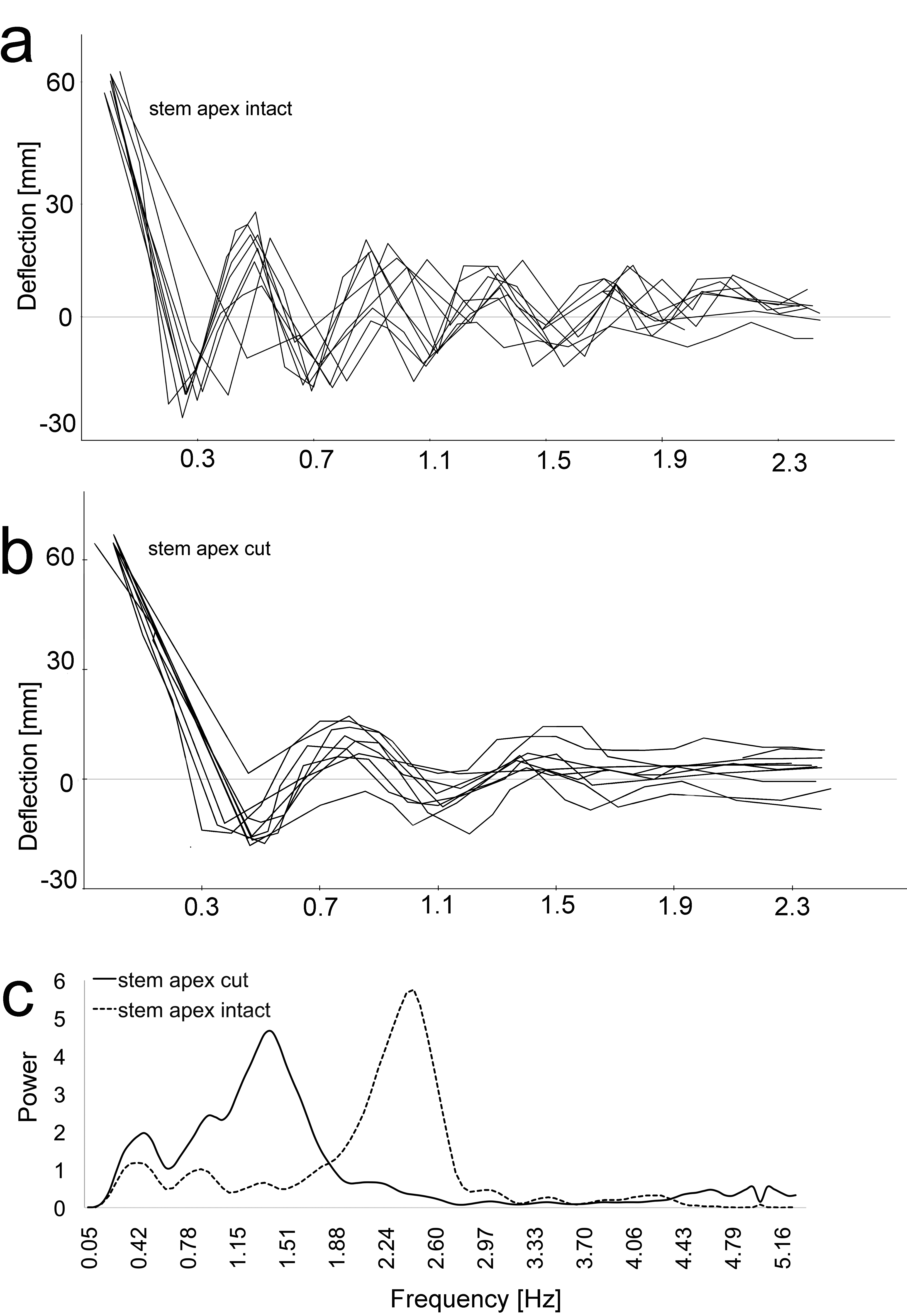

Supplement: Supplementary file 1 — Fig. S1 Comparison of the vibration of intact stems. Nine stems with the three top internodes cut off (a), and after static excitation via deflecting the stem by 20° from the vertical (b). Distribution of dominant frequencies presented in a periodogram for intact stems and apex cut stems (c) (TIFF 833 kb) [file 425_2017_2648_MOESM1_ESM.tif]

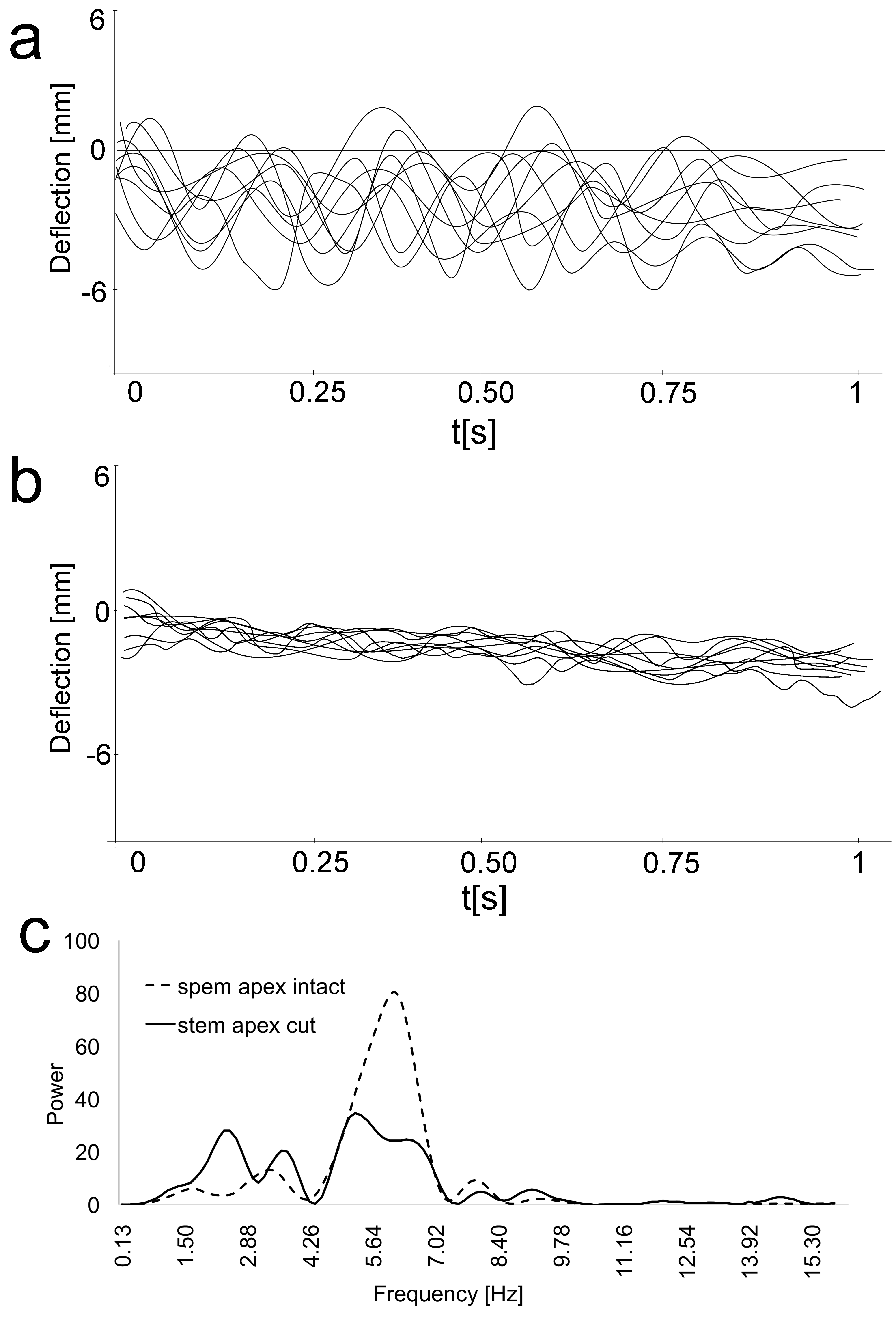

Supplement: Supplementary file 2 — Fig. S2 Comparison of the vibration of intact stems. Nine stems with the three top internodes cut off (a), recorded in wind tunnel (b). Distribution of dominant frequencies presented in a periodogram for intact stems and apex cut stems (c) (TIFF 1128 kb) [file 425_2017_2648_MOESM2_ESM.tif]
